# The influence of paternity leave uptake on parental post-partum depression: An ELFE cohort study

**DOI:** 10.1192/j.eurpsy.2023.798

**Published:** 2023-07-19

**Authors:** K. M. Barry, R. Gomajee, X. Benarous, M.-N. Dufourg, E. Courtin, M. Melchior

**Affiliations:** 1 ERES; 2 INSERM; 3 INED, Paris, France; 4 DLondon School of Hygiene and Tropical Medicine, London, United Kingdom

## Abstract

**Introduction:**

Many countries are currently expanding their paternity leave policies, which have positive effects on parental mental health.

**Objectives:**

We examined whether two weeks of paid paternity leave are associated with post-partum depression (PPD) in mothers and fathers at two months after the birth of their child.

**Methods:**

Data originated from The Etude Longitudinale Française depuis l’Enfance (ELFE) cohort study. A total of 10 975 fathers and 13 075 mothers with reported information on paternity leave and PPD at two months were included in the statistical analyses. Logistic regression models, using survey-weighted data and adjusted for confounders using Inverse Probability Weights (IPW), yielded Odds Ratios.

**Results:**

Fathers had a median age of 32∙6 (inter-quartile range (IQR) 36∙9 – 22∙6 years), and mothers had a median age of 30∙5 years (IQR 34∙0 – 27∙1 years) at the time of the ELFE child’s birth. Fathers who took paternity leave had reduced odds of PPD [0∙74 (95% CI: 0∙70 -0∙78)] as well as fathers who intended to take paternity leave [0∙76 (95 CI%: 0∙70 – 0∙82)] compared to fathers who did not take paternity leave. Mothers had an increased likelihood of PPD at two months if their partners took paternity leave [1∙13 (95 CI%: 1∙05 – 1∙20)]. Fathers’ educational level, work contract type nor the number of children in the family were found to be interactions (p>0.25).

**Conclusions:**

Taking and intending to take a two-week paid paternity leave is associated with lower odds of PPD in fathers. Mothers whose partners take paternity leave experience borderline higher odds of PPD at two months. Offering only a two-week paternity leave may protect fathers against PPD but does not significantly protect may increase mothers’ risk of against PPD onset.

**Disclosure of Interest:**

None Declared

